# NaPLeS: a natural products likeness scorer—web application and database

**DOI:** 10.1186/s13321-019-0378-z

**Published:** 2019-08-09

**Authors:** Maria Sorokina, Christoph Steinbeck

**Affiliations:** University Friedrich-Schiller, Lessingstrasse 8, 07743 Jena, Germany

**Keywords:** Natural products, Web application, Database, Docker container

## Abstract

Natural products (NPs), often also referred to as secondary metabolites, are small molecules synthesised by living organisms. Natural products are of interest due to their bioactivity and in this context as starting points for the development of drugs and other bioactive synthetic products. In order to select compounds from virtual libraries, Ertl et al. developed a natural product likeness score which was later published as an open data, open source implementation. Here we present NaPLeS, an easily portable, containerised, open source web application based on open data to compute natural product likeness scores for chemical libraries.

## Introduction

Natural products (NPs), often also referred to as secondary metabolites, are small molecules synthesised by living organisms. Natural products are of particular interest due to their bioactivity as they were optimised during evolution to have effective interactions with biological receptors. They are therefore considered as valuable starting points for the development of drugs and other bioactive synthetic products. In this context the evaluation of the compound NP-likeness is an important asset in the selection and optimisation of NP-like drugs and synthetic bioactive compounds. In 2008, Ertl and co-workers suggested a natural product (NP) likeness score, which, for a given molecule, is its similarity to the structure space covered by NPs compared to the structure space covered by synthetic molecules (SM). Such a score could be used to prioritise compounds from virtual libraries. Later, Jayaseelan and co-authors presented a purely open data, open source version of the NP-likeness scorer [1, 2]. Here we present an easily portable, containerised, open source web application based on open data to compute natural product likeness scores for chemical libraries.

The NP-likeness software suite NaPLeS consists of a web application that allows to compute the NP-likeness score online, of an easy to install local scorer to compute NP-likeness for big datasets and a MySQL database containing a large number of NPs from diverse public databases with pre-computed NP-likeness scores and other metrics. The web application is available at http://naples.naturalproducts.net and the source code can be obtained from GitHub (github.com/mSorok/NPdatabaseFiller and github.com/mSorok/NPlsWeb), the data and the data processor is available on Zenodo (10.5281/zenodo.2652372) and the NaPLeS web application is downloadable also on Zenodo (10.5281/zenodo.2652356). To the best of our knowledge, such a portable web application for private and public use is not yet available and this work therefore constitutes a significant improvement over the state of the art.

## Implementation

The implementation of the NP-likeness scorer web application and database is in two parts: the scorer training and the creation of the database and the development of the web application that connects on this database.

The NP-likeness score is based on the sum of the frequency of their fragments among NPs and SMs. Here the fragments are represented by atom signatures that are canonical circular descriptors of an atom’s environment in the molecule. The NP-likeness score is computed for each atom in a molecule represented as a directed acyclic graph, where every node is an atom and the edges are bonds between them. The levels of neighbourhood of an atom in a molecule is the height of the signature of that atom and determines the overall size of the fragment. In the present study atom signatures of height 2 were calculated. This height provides a better structural accuracy compared to height 1, and is not excessively large as height 3, which avoids over-training. A molecular signature is the sum of all its atom signatures.

### Training data

The training data was extracted and combined from several public and open databases: for natural products, it was integrated from ZINC, ChEBI [3], ChEMBL [4], PubChem [5], the Traditional Chinese Medicine DataBase (TCMDB [6]), NPAtlas [7], AfroDB [8], SANCDB [9], NuBBE [10], HIT [11], NPACT [12], StreptomeDB [13], UNPD [14], the manually curated data used for the study published in 2012 [2] and some other datasets not associated with any database or publication such as UEFS (accessed through ZINC). Datasets from companies, SelleckChem [15] and InterBioScreen [16], synthesising and selling the compounds were also used as they openly provide reliable molecular structures for natural products. The Super Natural II database [17] was excluded from the training dataset, due to uncertainty about its data quality and provenance (e.g. this database lists dodecahedrane, which is not an NP), but NP-likeness scores were computed for molecules stored in it and are displayed on the web application and in the MySQL database. Synthetic molecules were randomly selected from the ZINC database excluding all natural products, metabolites and other biogenic molecules. In total, the training set consists of 364,807 natural products and 489,780 synthetic molecules.

Cheminformatic processing in NaPLeS is realised with the Chemistry Development Kit (CDK) [18]. First, each molecule undergoes a curation process. The stereochemistry is removed from all molecules due to a big variation in databases of stereochemistry presence and depiction. This step is particularly important to avoid fragment redundancy. The molecule is then checked for disconnected parts and only the biggest one is kept for further curation. Molecules smaller than 6 atoms and containing non-organic atoms (allowed atoms: C, H, N, O, P, S, Cl, F, As, Se, Br, I, B, Na, Si, K, Fe) are discarded as suggested by Ertl et al. [1]. Then, redundant molecules between databases are eliminated based on their structural identity using their InChI. Next, linear and circular sugar moieties are removed from all molecules to omit moieties that are less distinctive due to their repetitive and redundant nature, albeit commonly present in NPs.

Atom signatures [19] (fragments) of height 2 are calculated for each molecule. For each fragment, its frequency among natural products compared to synthetic molecules is computed with Eq. 1, where NPi is the number of occurrences of the fragment *i* in natural products, SMi the number of occurrences of the fragment *i* in synthetic molecules, NPt is the total number of natural products and SMt is the total number of synthetic molecules. If the fragment is present several times in one molecule, its occurrence is counted accordingly (*e.g.* if the fragment occurs three times in one molecule, the total number of occurrences of this fragment in the corresponding molecule category will be increased by 3). The NP-likeness score of a molecule corresponds to the sum of frequencies of fragments in this molecule, corrected by its size (Eq. 2).

1 $$Frag_{i} = { \log }\left[ {\frac{{NP_{i} }}{{SM_{i} }}*\frac{{SM_{t} }}{{NP_{t} }}} \right]$$

2 $$NPls = \frac{{\mathop \sum \nolimits_{i = 0}^{N} Frag_{i} }}{N}$$

### NP-likeness database

The MySQL 5.8 Docker image is used to store the molecules, molecular fragments and the corresponding scores. The table ‘ori_molecule’ contains the information about the molecules from the public databases uploaded for the NP-likeness scorer training. Each molecule is described by the identifier from its original database, a SMILES, an InChIKey, the submission date, its original (i.e. source) database and its status (natural product, synthetic molecule or biogenic). In this table molecules can be redundant. The ‘molecule’ table contains unique fully connected molecules that are at least 6 atoms large and do not contain non-organic atoms (for definition, see above). Each molecule is associated with a unique identifier, its structural information (SMILES), whether it is a NP or not, if it contains sugar moieties, the NP-likeness scores computed for the molecule with and without the sugar moieties and various parameters such as the heavy and total atom counts (with and without the sugars), the number of rings, the number of repeated fragments (if 0 all fragments that constitute the molecule are found only once in it) and the number of predominant heavy atoms (carbons, oxygens and nitrogens). The tables ‘fragment_with_sugar’ and ‘fragment_without_sugar’ contain the atom signature of each fragment (SMILES-like notation), a unique fragment numerical identifier, the atom signature height (currently only the height 2 is stored) and the relative frequency of fragments in natural products computed with the Eq. 1. The table ‘molecule_fragment_cpd’ stores the relations between the fragment and molecule identifiers, whether it concerns fragments computed with sugar removal or not, and the number of occurrences of the fragment in the molecule. Two additional tables are required for the web application to run correctly: ‘user_uploaded_molecule’ and ‘user_uploaded_molecule_fragment_cpd’ that temporarily store the molecules submitted by the web application user and the information computed for them.

### Database filler

The code for the training described in the previous section is available in the NPdatabaseFiller application. This is an application containerised with Docker, running with Spring Boot and a MySQL database. The communication between the Java code and the MySQL database is handled by the Hibernate Object Relational Mapping (ORM).

NPdatabaseFiller uses the previously described training data and fills the MySQL database used by the NaPLeS web application. It can also be used as a stand-alone application to compute NP-likeness scores locally for a large number of molecules or to recreate the NP-likeness database from scratch.

**Fig. 1:**
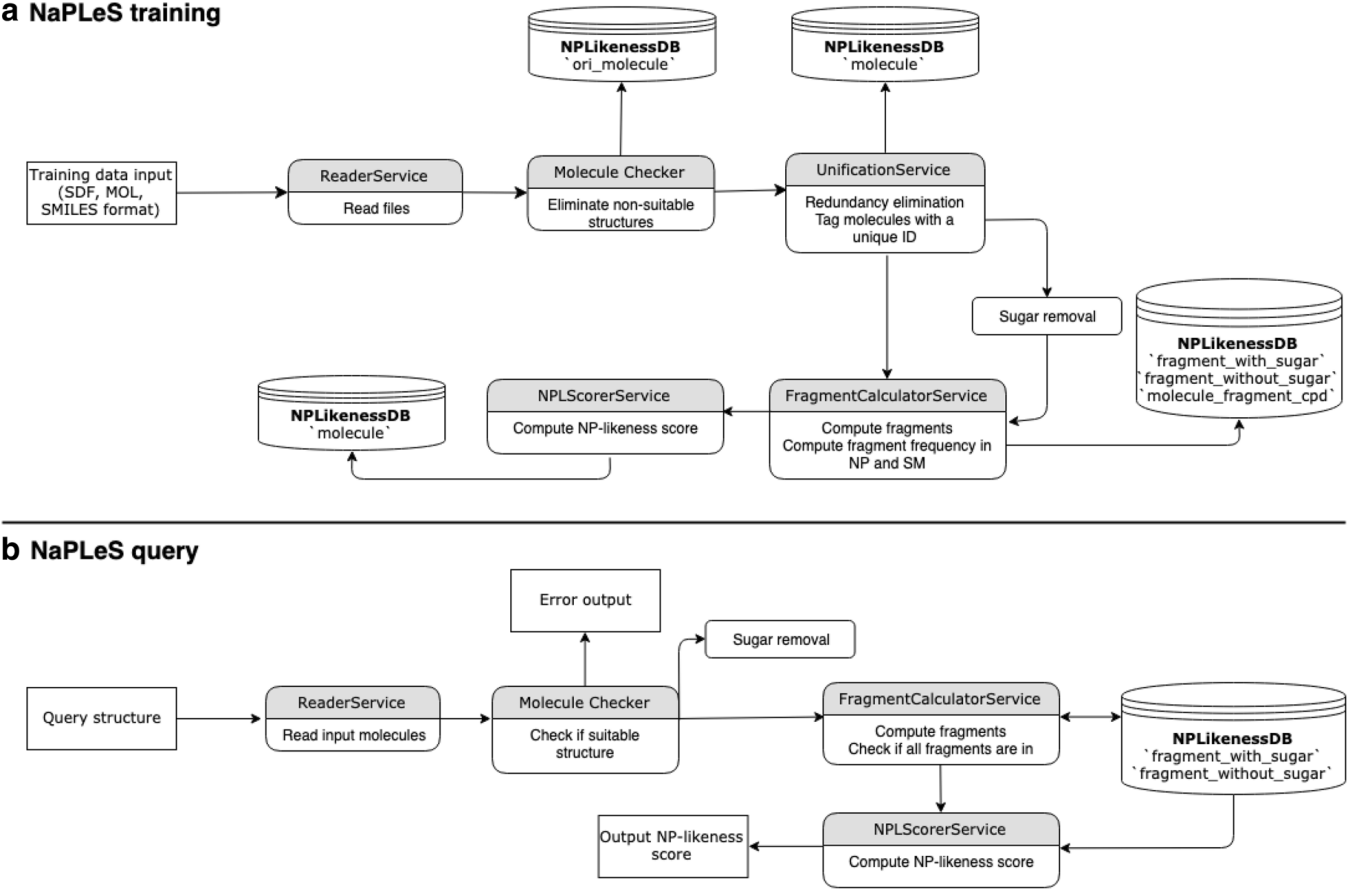
NaPLeS workflow schema. **a** NaPLeS training workflow schema. **b** NaPLeS query trough the web application

Three execution options for NPdatabaseFiller are available and can be selected by editing the docker-compose.yml file of the application. It is (a) possible to compute the NP-likeness scores from scratch for all submitted molecules. For this, it is necessary to provide molecular files with an appropriate format and an equivalent number of natural products and synthetic molecules for the training. To (b) compute the NP-likeness scores for one molecular file, without updating the whole database, and to (c) update the scores of all fragments in the database and the NP-likeness scores for all molecules present in the database. The last option is useful in case where a number of new molecules has been inserted in the database and the user wants to use them to re-train the scorer. A schematic presentation of the workflow is shown in Fig. 1a.

### Web application

The NaPLeS web application was developed with the Spring Boot framework and is composed of two Docker containers: the back- and the front-end build on an openjdk:8u171-slim image, and the database in a MySQL 5.8 container described in the previous section. The web application allows to compute the NP-likeness scores for submitted molecules from a big number of molecular fragments in a reasonable time (5 to 10 s for molecules with up to 20 heavy atoms, up to 20 s for molecules with more than 60 heavy atoms). The back-end is written in Java 8 using Spring Boot framework and relying on the Hibernate ORM for the communication with the MySQL database, and Thymeleaf as server-side Java template engine to serve dynamic content to the front-end.

Submission of molecules to compute their NP-likeness scores is possible in three ways: uploading a file in SDF, MOL or SMI (SMILES) format for a maximum of 200 molecules, pasting a SMILES string or drawing a molecule in the chemical editor. This threshold is defined only for the public instance of NaPLeS to allow a pleasant user experience and avoid long waiting times. It can be overwritten in a locally installed or cloud instance.

The molecular editor is using the OpenChemLib JavaScript libraries (https://github.com/cheminfo/openchemlib-js). The submitted molecules have their sugar moieties removed, then are fragmented in atom signatures, the scores of the matched fragments are retrieved, summed and normalised by the size of the molecule. The computed NP-likeness scores are reported in a results table with some additional molecular information, the depiction of the submitted molecules and if they exist, the identifiers of the submitted molecules in public databases. The results table is enhanced with DataTables.js library (https://datatables.net) and allows an easy export of tabular data in CSV and Excel formats, copying to the clipboard, sorting the results by all columns and a dynamic search. If any of the submitted molecules contains a fragment that is not in the database, the user is alerted, and the fragment is excluded from the score computation. In the results page distributions of NP-likeness scores as also depicted and where the computed results are situated among them. The schema of the NaPLeS query workflow is shown in Fig. 1b.

## Results

The original databases and the numbers of NP used for the NaPLeS’ NP-likeness scorer training are listed in Table 1. Data used for the training is exclusively open data. Note that Supernatural II database was excluded from the training set due to doubts on its content, but NP-likeness scores were computed for the molecules present in it and are available in the web-application.

**Table 1:** Size of individual data sets prior and after processing

Database	Number of parsed molecules	Number of unique molecules	Origin of molecules	Link/references
UEFS	503	478	Generalist	http://zinc.docking.org/catalogs/uefsnp
HIT	530	477	Plants	http://zinc.docking.org/catalogs/hitnp [11]
SANCDB	623	592	Plants	https://sancdb.rubi.ru.ac.za [9]
AfroDB	944	874	Plants	http://zinc.docking.org/catalogs/afronp [8]
Sellec Chem NP	1590	1411	Generalist	https://www.selleckchem.com/screening/natural-product-library.html [15]
NPACT	1572	1452	Plants	http://crdd.osdd.net/raghava/npact [12]
ChEMBL NP	1899	1328	Generalist	https://www.ebi.ac.uk/chembl [4]
NuBBE	2215	2022	Plants, Insects	https://nubbe.iq.unesp.br/portal/nubbe-search.html [10]
StreptomeDB	2443	2320	Bacteria	http://zinc.docking.org/catalogs/streptome [13]
PubChem NP	2938	2813	Generalist	https://pubchem.ncbi.nlm.nih.gov [5]
NANPDB	6840	3912	Generalist	http://african-compounds.org/nanpdb/ [20]
ChEBI NP	16223	15074	Generalist	https://www.ebi.ac.uk/chebi [3]
NPAtlas	20036	18909	Bacteria, Fungi	https://www.npatlas.org [7]
TCMDB	58388	50910	Plants	http://tcm.cmu.edu.tw [6]
InterBioScreen NP	67910	66789	Generalist	https://www.ibscreen.com/screening-compounds-download [16]
Manually curated dataset	77651	74368	Generalist	[2]
ZINC NP	85201	67320	Generalist	https://zinc15.docking.org/substances/subsets/natural-products
UNPD (via ISDB)	213206	157089	Generalist	http://oolonek.github.io/ISDB [14]
Super Natural II (not in the training set)	84554	59121	Generalist	bioinf-applied.charite.de/supernatural_new [17]

**Fig. 2:**
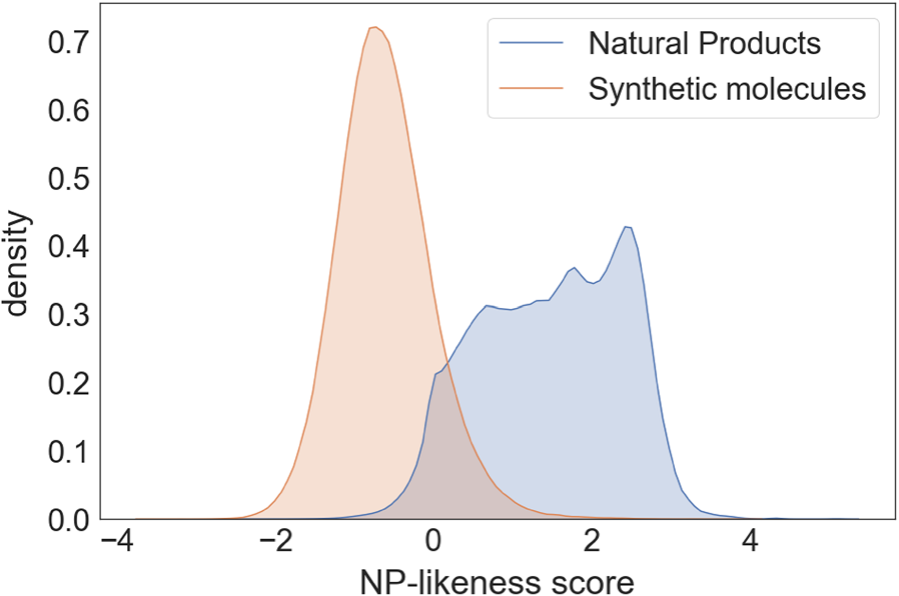
Distribution of the NP-likeness score for natural products and synthetic molecules

**Fig. 3:**
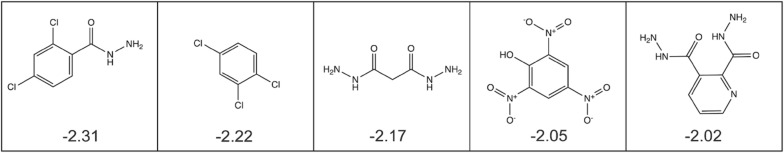
Top 5 molecules tagged as natural products in public databases with negative NP-likeness score. From left to right: 2,4-dichlorobenzohydrazide, 1,2,4-trichlorobenzene, malonohydrazide, picric acid and pyridine-2,3-dihydrazide

**Fig. 4:**
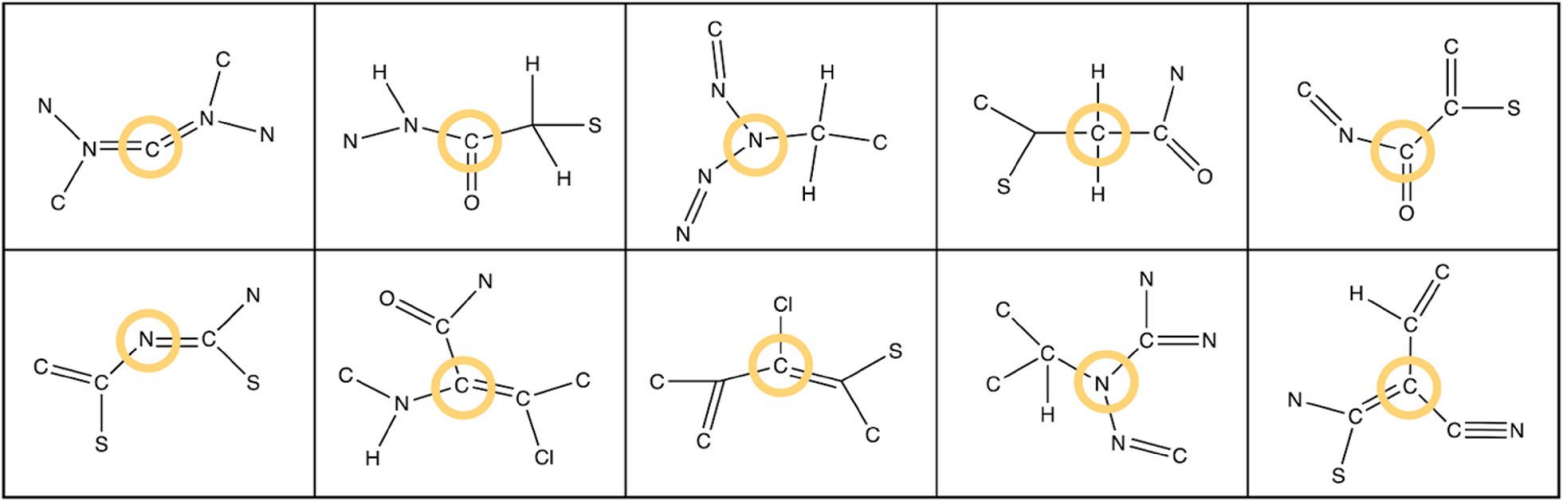
Top 10 fragments with highest frequency among synthetic molecules

**Fig. 5:**
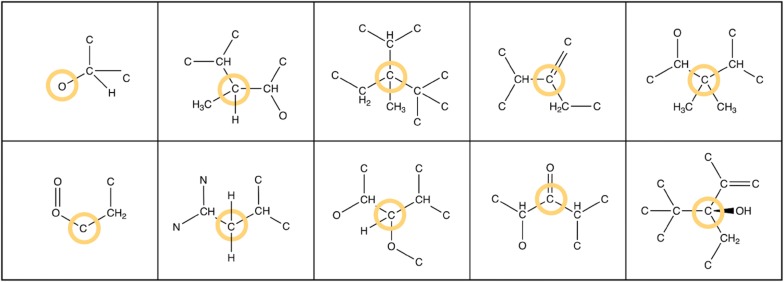
Top 10 fragments with higher frequency among natural products

**Fig. 6:**
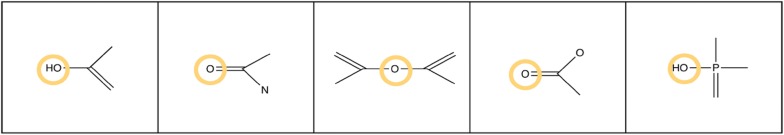
Examples of highly repeated in natural products fragments centered on an oxygen

At the time of publication of this article, NaPLeS works with a total of 364,807 unique natural products and 489,780 unique synthetic molecules, unified on the basis of structural identity ignoring stereochemistry. The minimal NP-likeness score for this dataset is − 3.63, the maximum is 5.15 and the global average is 0.27 (Fig. 2). The score for synthetic molecules ranges between − 3.61 and 4.01 with an average of − 0.61. The score for natural products is distributed between − 2.31 and 5.15 with an average of 1.48. The top 5 molecules tagged as a NP in public databases with the most negative NP-likeness scores are shown in Fig. 3. The most common fragments in NPs and SMs have different structures and atomic composition (Figs. 4, 5). 95% of molecules in our dataset (NP and SM) have at least one repeated fragment that is not centred on a hydrogen (the latter are not informative as most of fragments centered on a hydrogen in any molecule will be repeated). The biggest difference between NPs and SMs in terms of repeated fragments is the dominant presence of repeated fragments centred on an oxygen in NP compared to SM: 65% of NPs with at least one oxygen have at least one repeated fragment centred on an oxygen (Fig. 6) and only 38% of SM with at least one oxygen possess such fragments. This difference is lesser, although remarkable, for molecules containing nitrogen: fragments centred around a nitrogen are repeated in 14% of NPs compared to 8% of SMs.

## Discussion

The described software suite is the update and improvement of the NP-likeness score published in 2012 by Jayaseelan et al. [2]. This previous version was available for download as a Jar executable and a Taverna workflow. We believe that our containerised update is easier to use for researchers not accustomed to Unix or Windows command-line.

The NP-likeness score can be also used to identify molecules mistakenly annotated as natural products in databases. Indeed, in Fig. 3 are shown the top 5 molecules annotated as NP with the lowest NP-likeness scores. Manual examination of these molecules shows that all of them are in reality synthetic and have been erroneously tagged as NP due to their direct interaction with living organisms (three of them are drugs, one is a bio-remediable toxin and one is a derivate from a natural product). Despite their presence in the training set, their non-natural product likeness was not affected. However, this point also shows that a more extensive curation of the NP dataset is needed. Therefore, NaPLeS’ NP-likeness database will be regularly updated and curated for better data quality.

NP-scout [21] is, to our knowledge the only other web application allowing to compute an estimation on how much a submitted compound is likely to be a natural product. The approach used in NP-scout is different, as it computes a probability of a molecule to be a NP based on its physico-chemical properties, Morgan2 fingerprints and MACCS keys. Similarly, to the NP-likeness scorer presented here, NP-scout is also trained on public data, and has a similar processing time per submitted molecule (from 3 to 20 s depending on the molecule size). The NP-likeness scorer accepts more file formats, its results are simpler to interpret and there are more output options. Both tools are consistent with reality and can be seen as complementary, however, NP-scout has a more complex approach, that is less straightforward to understand and to interpret.

## Conclusions

Here we have presented NaPLeS, an easily portable, containerised, open source web application based on open data to compute natural product likeness scores for chemical libraries. The NaPLeS NP-likeness scorer is available as a containerised web application and a containerised standalone version whose sole requirement is a docker installation. It is a useful tool that can help to guide the design of new molecules toward interesting regions of chemical space which have been identified as active regions by natural selection. The compilation of a big number of NP with their characteristics and pre-computed fragments offered in the NP-likeness database facilitates the exploration of the structural features of NP on a large scale.

Future developments will focus on the visualization of fragments in the query molecule as well as the search of natural products and their scores in the NP-likeness database by molecular fragments. It is also planned to include the NP-likeness score computed on molecules without the sugar removal to the results’ visualisation to allow the users to estimate by themselves the importance of the sugar moieties in the submitted molecules. A REST API, allowing interaction with NaPLeS without browser will be added to the application in the near future.

### Availability and requirements

- Project name: NaPLeS
- Project home page: https://naples.naturalproducts.net
- Operating system(s): Platform independent
- Programming language: Java
- Other requirements: Docker
- License: MIT
- Any restrictions to use by non-academics: no.

## Data Availability

Data and software are freely available under the MIT license. The web application is available at http://naples.naturalproducts.net and the source code can be freely obtained from GitHub (github.com/mSorok/NPdatabaseFiller and github.com/mSorok/NPlsWeb), the data and the data processor is available on Zenodo (10.5281/zenodo.2652372) and the NaPLeS web application is downloadable also on Zenodo (10.5281/zenodo.2652356).
